# Association between haematological values and heat shock protein 70 of sickle cell disease patients in Ado-Ekiti, Ekiti State, Nigeria

**DOI:** 10.11604/pamj.2022.43.47.33346

**Published:** 2022-09-29

**Authors:** Ayodeji Olusola Dickson Olayanju, Adedoyin Adeleke, Chisara Sylvestina Okolo, Ogunyemi Omotomilola Ogunyemi, Ogunjobi Kemisola Mary

**Affiliations:** 1 Department of Medical Laboratory Science, Afe Babalola University, Ado Ekiti, Nigeria

**Keywords:** Sickle cell disease, heat shock protein 70, haematological parameter

## Abstract

Sickle cell disease, a genetically inherited blood disorder is a major cause of mortality and morbidity in Nigeria. This condition has significant pathological consequences that result in hemolytic events, induction of inflammatory process, vaso-occlusive episodes, and the stress response that leads to the induction of heat shock protein (HSP) 70. Therefore, this study aimed at correlating the level of serum heat shock protein 70 to haematological parameters in sickle cell subjects. A total of eighty-eight (88) consented participants were recruited for this study, which included apparently healthy persons with homozygous hemoglobin (HbAA 20), heterozygous hemoglobin (HbAS 30), homozygous hemoglobin (HbSS 30), and homozygous hemoglobin (HbSC 08). Subjects are in crisis and steady state. Venous blood samples (5 mls) were collected from subjects in ethylene diamine tetra acetic acid (EDTA) container and analyzed hemoglobin variants using hemoglobin electrophoresis, HSP 70 by Elisa method, and full blood count using standard methods. We demonstrated a significant increase (P<005) in HSP 70 levels of sickle cell disease HbSS and HbSC in steady state and crises when compared to the controls HbAA and HbAS. A significant (p<0.0001) increase noticed in the crisis state is higher than in the steady state. While the mean value of mean corpuscular hemoglobin concentration (MCHC) (35.1±43.4), pack cell volume (PCV) (22.4±2.7), hemoglobin (Hb) (8.8±0.9), absolute neutrophil count (386.4±31) and Absolute neutrophil count (7.0±2.1) in steady state subjects was significantly higher (p<0.01), as compared to crisis state (29.5±2.5, 21.8±3.4, 7.3±1.8, 269.5±42 and 6.5±2.5) for the respective parameters, whereas, mean corpuscular volume (30.5±3.1), white blood cell (16.8±3.4), absolute lymphocyte count (5.0±1.3) in sickle cell disease subject in crisis state are significantly higher (p<0.01) than in steady state (29.3±2.2, 11.3±2.8, 4.3±1.1) respectively. The mean value of mean corpuscular volume (87.3±8.2) in the crisis state was higher when compared to the steady state (83.5±7.2) and the mean value of red bloood cell (2.7±0.4) in the steady state was higher when compared to the crisis state (2.3±0.7). The differences were not significant (p<0.01). These findings suggest that an association exists between Hsp 70 and haematological parameters in sickle cell subjects. This implies that Hsp 70 might be a marker in oxidative stress, hypoxia, vaso-occlusion crisis, and increased serum Hsp 70 levels seem to reflect systemic inflammation. However, further studies are required to determine whether circulating Hsp 70 plays a causative role in the pathogenesis of sickle cell.

## Introduction

Sickle cell disease is an inherited genetic disease that is characterized by vaso-occlusion, ischemia and hemolysis, and chronic anaemia. The primary pathophysiological alteration of red blood cells has countless consequences, including the production of inflammatory molecules [1]. The complex pathophysiology of this genetic disease involves abnormal activation of coagulation cascade and fibrinolysis, evidenced by the increased risk of thrombotic manifestations [2]. Whenever there is frequent stress on cells of sickle cell patients due to inflammation, oxidative stress, hypoxia, and vaso-occlusion, there is an evolutionarily stress-induced molecule called heat shock that is ubiquitously expressed in all eukaryotic cells [3]. Heat shock protein also play an important role in protein stabilization such as assembling of multi-protein complexes, folding or unfolding of proteins, transport or sorting of proteins into correct compartments at the subcellular level, control of cell-cycle and signalling, as well as cell protection against stress such as nutrient deficiency, oxidative stress [4]. The release of HSP70 to the extracellular matrix is triggered by cell stress and the accumulation of the chaperone in extracellular space serves as a dangerous signal to the immune system [5]. Baseline sickle cell disease (SCD) subjects have significantly higher circulating serum Hsp70 levels as compared with normal controls and patients with vaso-occlusive crisis (VOC) and have significantly higher circulating serum Hsp70 levels as compared with steady-state SCD [1]. Heat shock protein 70 levels in the blood are higher in patients with peripheral and renal vascular disease, Fehrenbach *et al*. [6] have also reported higher Hsp70 levels in the blood of patients with prostate cancer and during and after exercise respectively. Heat shock protein levels in the blood were also found to be higher in patients with heart failure [7], as well as in children with septic shock [8], after surgical procedures. In pregnant women, the Hsp70 level was reported to be considerably lower than healthy non-pregnant women [9]. Furthermore, it has been demonstrated that serum Hsp70 levels are significantly higher in patients with sickle cell disease and increase further during VOC, suggesting that circulating serum Hsp70 might be a marker for VOC in SCD [1]. However, much work is yet to be published on the correlation between haematological values and heat shock protein 70 in sickle cell disease patients, and the clinical implications of elevated circulating Hsp70 levels in sickle cell patients are uncertain at this time. Therefore, the present study aimed to examine if the levels of serum heat shock protein 70 correlate to haematological parameters in sickle cell subjects.

**Objectives:** i) to determine and compare the serum heat shock protein in individuals with hemoglobin AA, hemoglobin AS and sickle cell patient (hemoglobin SS); ii) to determine and compare the serum heat shock protein in sickle cell patients in crisis and sickle cell patients in steady state; iii) to determine if there is a correlation or relationship between HSPs and Haematological parameters in SCD and control subjects.

**Null hypothesis (H0):** there is no correlation relationship between the levels of heat shock proteins and hematopathology occurring in sickle cell disease.

**Alternate hypothesis (H1):** there is a correlation relationship between the levels of heat shock proteins and hematopathology occurring in sickle cell disease.

## Methods

**Study design and setting:** this is a cross-sectional study which consisted of 30 HbSS patients, 8 HbSC patients, 20 Hb AA and 30 HbAS subjects as the control group. This was a study of steady-state sickle cell anaemia patients seen in the two tertiary hospitals in Ekiti-State (Federal Teaching Hospital, Ido Ekiti and Abuad multisystem hospital). The controls were recruited from apparently healthy children, whose Haemoglobin phenotypye were AA and AS. Hemoglobin SS (case) in steady state was outlined by the absence of crisis within the preceding four weeks and intro mission within the preceding six months. Hemoglobin phenotypes of subjects was determined by cellulose acetate hemoglobin electrophoresis at pH 8.6 using tri-buffer. According to 2016 census figure, the population of the 16 local government is 2,398,957 and the two tertiary hospital are serving them. The state is mainly an upland zone, rising over 250 meters above area level. It coordinates is 7°40´N 5°15´E. The individuals in Ekiti State are preponderantly Yoruba and largely farmers by occupation.

**Inclusion criteria:** sickle cell anaemia confirmed status patients, the age limit of 1 to 15 years. Subjects were not transfused with blood in the last six months and not in crisis in the last four weeks.

**Exclusion criteria:** subjects that were hemoglobin AC or CC genotype and those with homozygous SS haemoglobin genotype that had other underlying medical conditions.

**Control group:** apparently healthy subjects with haemoglobin AA and AS genotype.

**Data collection:** questionnaires were used to get biodata and the tests mentioned below were used to get the remaining data; b) data collection was done primarily by administering of a structured questionnaire to each volunteer. Furthermore, the majority of data used in this research were from the different test results. Questionnaires were used to get biodata and the tests mentioned below were used to get the remaining data.

**Bias:** we did proper planning and evaluation of hypothesis and methods. Review of our research was done outside our team members, then, after the results were communicated to the volunteers.

**Sample size calculation:** the minimum sample size was calculated by single proportion formula based on a 2% prevalence of sickle cell disease in Nigeria [10].


$$N_{\circ }=\frac{Z^{2}pq}{d^{2}}$$

Where, No= Minimum sample size, Z^2^= abscissa of the normal curve that cuts an area at the tails (equals the desired confidence levels at 95%), d = degree of uncertainty (Allowance for error = 0.05), P = estimated portion of an attribute that is present in the population (prevalence of sickle cell in Nigeria 2%) q = 1-p [11].


$$N=\frac{1.96^{2}\times 0.02\times (1-0.02)}{0.05^{2}}$$

$$N=\frac{3.8416\times 0.02\times 0.98}{0.0025}=30.1$$

**Sampling:** the method of sampling employed in this study is simple random sampling. Blood samples were collected under aseptic conditions. Five (5ml) of blood sample was collected into EDTA bottle for full blood count analysis and Haemoglobin electrophoresis. The plasma from the EDTA bottle was used for heat shock protein analysis.

**Laboratory analysis:** procedures of the test needed, haemoglobin electrophoresis; buffer A (Anode) tris (hydroxymethyl-aminomethane)..25.2g, EDTA (ethylene diamine tetra-acetic acid)..2.5g, boric acid..1.9g, water to..1000ml

**Buffer B (cathode):** sodium diethyl barbiturate diethyl barbituric acid..5.15g, diethyl barbituric acia..0.92g, water to ..1000ml

**Method:** the cellulose acetate membrane strip was soaked in a mixture of buffer A and buffer B together with saponin (0-1%). One 1 ml of 10% saponin was added to 100 ml of a 50-50 mixture of buffer A and buffer B. The strip was blotted and applied to the bridge of the tank. A thin line of whole blood was applied at the midpoint of the strip. The lid was put on and the samples were allowed to soak into the cellulose acetate membrane strip. The red cells were lysed by saponin. After three to five minutes the current was switched on (14 mA at 200 V). The haemoglobin bands move to the cathode. The individual bands were separated and interpreted.

**A full blood count was carried out using mindray BC 5000 on whole blood samples as follows:** the blood in the EDTA bottles was properly mixed. The sample number was entered. The stopper was opened. The tube was set to the sample prop and in that condition, the start switch was pressed. The tube was held in that position until the buzzer sound “beep, beep” was heard two times and the liquid-crystal display (LCD) screen displayed “analyzing” before the tube was removed. The unit then executes automatic analysis and displays the result on the LCD screen.

**Serum heat shock protein assay:** the plasma harvested from the whole blood sample was dispensed into plain bottles for heat stock protein analysis using enzyme-linked immunosorbent assay (ELISA), elabscience kit.

**Procedure:** all materials were assembled and the reagents were prepared at room temperature before use. Excess microplate strips were removed from the plate frame and were returned to the foil pouch containing the desiccant pack, resealed and returned to 4°C storage. Fifty (50) µL of all samples or standards were added to microplate wells. Fifty (50) µL of the antibody cocktail was added to each microplate well, the plate was sealed and incubated for 1 hour at room temperature on a plate shaker set to 400 rpm. The microplate wells were washed with 3x350 µL 1X wash buffer. It was washed by decanting from wells then 350 µL 1X wash fuffer PT was dispensed into each well. After the last wash, the plate was inverted and blotted to remove excess liquid. Hundred (100) µL of tetramethylbenzidin (TMB) substrate was added to each microplate well and incubated for 10 minutes in the dark on a plate shaker set to 400 rpm. Hundred µL of stop solution was added to each microplate well. The well was placed on a plate shaker for 1 minute to mix, 450 nm optical density was used in reading the microplate. This was the endpoint reading.

**Statistical analysis:** the statistical analysis was done using SPSS version 16.0 software package and Excel spreadsheet software (Microsoft office, 2016). Correlation analysis was done to test the association between parameters while student´s t-test and ANOVA were used to compare the means.

**Missing data:** deletion approach was applied to missing data.

**Interpretation of analysis:** this was done using statistical analysis and calculation of means, student's t-test, standard deviation, confidence intervals, and P values of the samples and was determined using standard statistical software

**Ethical considerations:** ethical approval was obtained following the Helsinki declaration from the Health Research Ethical Committee, Afe Babalola University, Ado Ekiti (Protocol No: ABUADREC/28/03/2020/01). The study participants were enlightened concerning the aim of the study and written consent was obtained from every participant before sampling was done.

## Results

**Figure 1:**
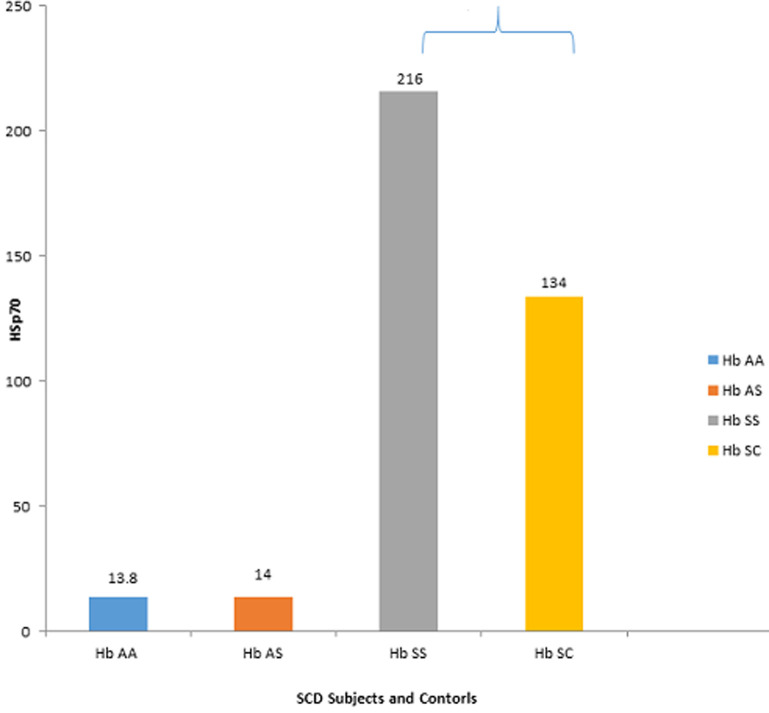
sickle cell disease subjects and their controls/heat shock protein 70

**Figure 2:**
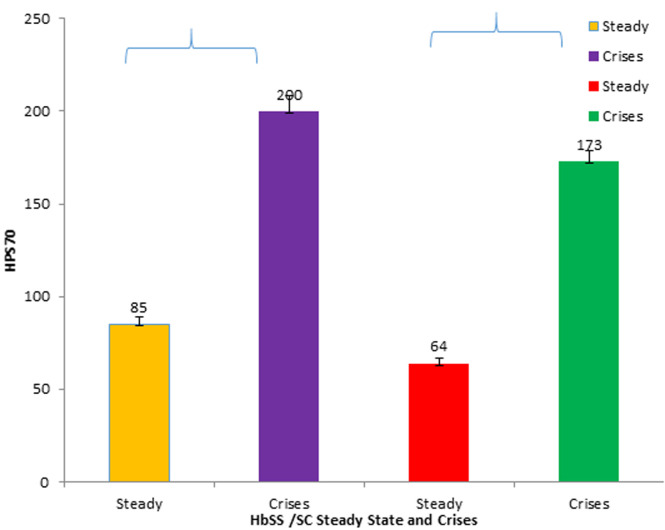
mean value of heat shock protein 70± standard deviation in SS and HbSC subjects (steady state and crises)

**General characteristics of study population:** a total of 88 subjects were examined in this study which was predominated by subjects whose age groups fell between 11-15 years and mean age (8.28±4.28). The percentage proportion of males to females is 54.5%. The distribution of ethnic groups of volunteers in this western part of Nigeria was majorly the Yoruba's with the highest population of 68.2% followed by the Igbos (20.5%) and Hausa's (11.4%) Table 1. The mean value of HSP70 in homozygous haemoglobin AA (HHAA) (13.8±1.7), heterozygous haemoglobin AS (HHAS) (14.0±1.2), homozygous haemoglobin SS (HH SS) (216±3.2), homozygous haemoglobin (134±2.1), and mean value of HSP70 in SCD subject (HbSS and HbSC) was significantly higher (p<0.05) when compared to the control HbAA and HbAS (Figure 1). The mean value of Hsp70 in SCD subjects (HbSS and HbSC) in crisis state (200±8.3 and 173±5.8 respectively) is significantly higher (P<0.0001) than in steady state (85±4.1 and 64±2.9 respectively) (Figure 2).

**Table 1:** general characteristics of study population

Variables	Frequency	Mean	Standard deviation
**Age group**			
01-05	29	8.28	4.28
06-10	25	8.28	4.28
11-15	34	8.28	4.28
**Tribe**	**Frequency**	**Percentage**	
Igbo	18	20.5	
Hausa	10	11.4	
Yoruba	60	68.2	
**Sex**			
Male	48	54.5	
Female	40	45.5	

**Relationship between haematological parameters and sickle cell disease states:** mean value of MCHC (35.1±3.4), pack cell volume (22.4±2.7), Hb (8.8±0.9), platelets (PLT) (386.4±31) and absolute neutrophil count (7.0±2.1) in SCD. Subjects in steady state were significantly higher (p<0.01) than in crisis state (29.5±2.5, 21.8±3.4, 7.3±1.8, 269.5±42, and 6.5±2.5) for the respective parameters whereas, mean corpuscular haemoglobin (30.5±3.1) white blood cell (16.8±3.4), absolute lymphocyte count (5.0±1.3) in SCD subject in crisis state are significantly higher (p<0.01) than in steady state (29.3±2.2, 11.3±2.8, 4.3±1.1) respectively. The mean value of mean corpuscular volume (87.3±8.2) in the crisis state was higher when compared to the steady state (83.5±7.2) and the mean value of red blood cell (2.7±0.4) in the steady state was higher when compared to the crisis state (2.3±0.7). The differences were not significant (p<0.01)

**Correlation between Hsp70 and haematological parameters in sickle cell disease subjects:** a weak positive correlation relationship exists between Hsp70 and MCV (r=0.13), mean corpuscular haemoglobin (r=0.07), white blood cell (r=0.38), platelets (r=0.01) and absolute neutrophil count (r=0.28) with R-value greater than 0 and less than or equal to 0.3 (r = 0 < r ≤ 0.3). A weak negative linear relationship also exists between Hsp 70 and mean corpuscular haemoglobin concentration (r = -0.21), pack cell volume (r= -0.12), haemoglobin (r= -0.20), absolute lymphocyte count (r = -0.21) and red blood cell (r= -0.20) with R-value lesser than 0 and less than or equal to -0.3 (r ≤ 0.3) as seen in (Table 2).

**Table 2:** heat shock protein 70 correlation between sickle cell disease and control subjects

Parameters	Sickle cell disease	Control
Mcv (fl)	0.13	0.41
Mch (pg)	0.07	0.40
Mchc (g/dl)	-0.21	0.11
Pcv (%)	-0.12	0.12
Hb (g/dl)	-0.20	0.09
Wbc (10^9^/l)	0.38	0.26
Platelet (10^6^/l)	0.01	0.22
Anc (cell/ul)	0.28	0.51
Alc (cell/ul)	-0.21	0.05
Rbc (10^12^/l)	-0.20	0.02

MCV: mean corpuscular volume; MCH: mean corpuscular haemoglobin; MCHC: mean corpuscular hemoglobin concentration; PCV: pack cell volume; HB: hemoglobin; WBC: white blood cell; ANC: absolute neutrophil count; ALC: absolute lymphocyte count; RBC: red blood cell

**Correlation between HSP 70 and haematological parameters (r value) in control subject:** shows a moderate positive linear relationship exists between HSP 70 and mean corpuscular volume (r = 0.41), mean corpuscular haemoglobin (r = 0.40) and absolute neutrophil count (r = 0.51) with R-value greater than 0.3 and less than or equal 0.5 > 0.3 r ≤ 0.5). A weak positive correlation relationship between HSP70 and platelets (r = 0.22), mean corpuscular haemoglobin concentration (0.11), pack cell volume (r = 0.12), haemoglobin (r = 0.09), red blood cell (r = 0.02) and white blood cell (r = 0.26) and absolute lymphocyte count (r = 0.05) with R-value greater than 0 and less than or equal to 0.3 (r = 0 < r ≤ 0.3).

## Discussion

This study was designed to determine the level of HSP 70 in sickle cell and control subjects, and the relationship between heat shock and haematological parameters. The mean value of the packed cell volume (PCV) in steady state (22.4±2.7) and red blood cell count (RBC) (2.7±0.7) were significantly reduced. This may be due to an increase in damage to red cells and hemolysis that occurs during crises in sickle cell subjects [12] and shortened the red cell survivor. This result contradicts the study done at the University of Benin Teaching Hospital in South-south Nigeria [13]. The variation in this study could be due to a larger sample population in the comparison study. The PCV and RBC value in SCD subjects has a weak negative correlation with HSP70 and a weak positive correlation with both controls. There was a weak negative correlation between HSP70 and hemoglobin (HB) in SCD subjects and a weak positive correlation in the control subjects. The mean haemoglobin value obtained in the steady state (8.8±0.9) is increased compared to the crisis state (7.3±1.8). This agrees with the study done in the department of pediatrics, haematology/oncology Unit, Amadu Bello University Teaching Hospital North-West Nigeria [14]. The lower HB value in steady state in this study is attributed to chronic hemolysis [15] which is the feature of SCD crisis and low erythropoietin response [16]. Mean cell hemoglobin concentration (MCHC) showed a weak negative linear correlation with HSP 70 in SCD and a weak positive correlation in the control. Steady-state subjects had a mean value of (35.1±3.4) which was higher than that of the crisis state (29.5±2.5). However, this is not the case in the study of Omoti, [13], where the mean value of the crisis state was higher than the steady state. This study reveals the mean value of the MCV in the steady state (29.3±2.2) mean cell volume (83.5±7.2) was lower than that of the crisis state MCV (30.5±3.1) and MCH (78.3±8.2). This result may be due to SCD being a chronic hemolytic state that stimulates hematopoiesis and hemopoietic activities during VOC. This is in agreement with the study of Omoti [13]. A weak positive correlation was observed between HSP 70, MCH and MCV in SCD subjects and a moderate positive correlation with both controls.

The relationship between Hsp 70 and platelet count showed a weak positive correlation in sickle cell disease subjects and also control subjects. The mean value in the steady state (386.4±31) is increased compared to the steady state (269.5±42). However, this was not the case in the study by Yakubu [14]. This result may be due to the fact that the negative feedback effect on erythropoietin production in SCD subjects as a result of anaemia could be responsible for thrombocytes. Reduce or absent splenic sequestration of platelet as a result of hypersplenism in sickle cell disease [17]. This study found white blood cell count to have a weak positive correlation with HSP70 in both SCD subjects and control subjects. The mean value obtained from the steady state (11.3±2.8) was lower when compared to the crisis state (16.8±3.4) which is in agreement with Omoti [13]. The higher value can be associated with auto-splenectomy resulting from recurrent splenic vesicle occlusion which makes patients more vulnerable to overwhelming infection and hence high WBC count [18]. Absolute neutrophil count shows a weak positive correlation with HSP70 in SCD patients and a moderate positive with the control. In the steady-state subject, the mean value (7.0±2.1) is higher than that of the crisis state (6.5±2.5). There was a weak negative correlation between HSP70 and absolute lymphocyte count in SCD patients and a weak positive correlation with the control subjects. The mean value in the steady state (4.3±1.1) was lower compared to the crisis state (5.0±1.3).

**Interpretation:** since HSP 70 has been shown to prevent the aggregation of damaged proteins and assist in the assembly of polypeptides of newly synthesized proteins, this implies that HSP70 might be a marker in oxidative stress, hypoxia, vaso-occlusion crises and increased serum HSP70 levels seem to reflect systemic inflammation.

**Limitations:** due to time constraints, larger samples would have been used and lack of funds to execute the larger samples. Limited documented information on the subject matter is a huge challenge. The ability of all participants to give their consent and be willing to give 5mls of blood sample is an advantage.

## Conclusion

It was observed that HSP 70 was higher in sickle cell disease subjects, and also the increase was observed to be higher in the crisis state than in the steady state. These findings suggest that an association exists between HSP 70 and haematological parameters in sickle cell subjects. This implies that HSP70 might be a marker in oxidative stress, hypoxia, vaso-occlusion crisis, and increased serum HSP70 levels seem to reflect systemic inflammation. However, further studies are required to determine whether circulating HSP70 plays a causative role in the pathogenesis of sickle cell.

### What is known about this topic

- Heat shock proteins (HSPs) belong to the family of conservative polypeptides with a high homology of the primary structure and are essential for development and cellular function;
- Heat shock proteins 70, functions as a cytosolic chaperone involved in facilitating protein folding, degradation, complex assembly, and translocation; these functions have been shown to prevent the aggregation of damaged proteins and assist in the assembly of polypeptides of newly synthesized proteins;
- They might be a marker for vaso-occlussive crises in sickle cell patients.

### What this study adds

- In sickle cell disease subjects in a crisis state, Heat shock protein 70 values are increased than in a steady state;
- This is a pointer that there is an association between Heat shock protein 70 and haematological parameters in sickle cell subjects;
- This implies that Heat shock protein 70 might be a marker in oxidative stress, hypoxia, and vaso-occlusion crises.
